# Invasive Species and Biodiversity Crises: Testing the Link in the Late Devonian

**DOI:** 10.1371/journal.pone.0015584

**Published:** 2010-12-29

**Authors:** Alycia L. Stigall

**Affiliations:** Department of Geological Sciences and OHIO Center for Ecology and Evolutionary Studies, Ohio University, Athens, Ohio, United States of America; Paleontological Institute RAS, United States of America

## Abstract

During the Late Devonian Biodiversity Crisis, the primary driver of biodiversity decline was the dramatic reduction in speciation rates, not elevated extinction rates; however, the causes of speciation decline have been previously unstudied. Speciation, the formation of new species from ancestral populations, occurs by two primary allopatric mechanisms: vicariance, where the ancestral population is passively divided into two large subpopulations that later diverge and form two daughter species, and dispersal, in which a small subset of the ancestral population actively migrates then diverges to form a new species. Studies of modern and fossil clades typically document speciation by vicariance in much higher frequencies than speciation by dispersal. To assess the mechanism behind Late Devonian speciation reduction, speciation rates were calculated within stratigraphically constrained species-level phylogenetic hypotheses for three representative clades and mode of speciation at cladogenetic events was assessed across four clades in three phyla: Arthropoda, Brachiopoda, and Mollusca. In all cases, Devonian taxa exhibited a congruent reduction in speciation rate between the Middle Devonian pre-crisis interval and the Late Devonian crisis interval. Furthermore, speciation via vicariance is almost entirely absent during the crisis interval; most episodes of speciation during this time were due to dispersal. The shutdown of speciation by vicariance during this interval was related to widespread interbasinal species invasions. The lack of Late Devonian vicariance is diametrically opposed to the pattern observed in other geologic intervals, which suggests the loss of vicariant speciation attributable to species invasions during the Late Devonian was a causal factor in the biodiversity crisis. Similarly, modern ecosystems, in which invasive species are rampant, may be expected to exhibit similar shutdown of speciation by vicariance as an outcome of the modern biodiversity crisis.

## Introduction

The Late Devonian (Frasnian-Famennian) interval is traditionally considered to rank among the “Big Five” mass extinction events in Phanerozoic history with peak diversity decline in the late Frasnian Stage [1]–[3]. Certainly this interval included a dramatic biodiversity crisis during which high levels of ecosystem reorganization occurred [4]. Its status as a “mass extinction”, however, has been questioned based on statistical analyses of extinction rates; Late Devonian extinction rates are not significantly higher than extinction rates during background intervals [3], [5].

Biodiversity loss can occur due to either (or both) elevated extinction levels or reduced speciation rates. Because the role of elevated extinction appears to be minimal during the Late Devonian Biodiversity Crisis, an analysis of speciation dynamics across this interval can provide critical insight into the faunal dynamics of this key interval in life history. Indeed, reduced speciation rates during the Frasnian crisis interval have been recognized since at least 1989 [1], [2], [4], [6]. However, neither the statistical significance of this reduction nor its causes have been previously explored using phylogenetically constrained species-level data or within an analytical framework that could assess speciation mode.

Previous attempts to examine Late Devonian biodiversity dynamics have focused on either on either ecosystem level patterns [4] or analyses of genus and family level data extracted from global taxonomic databases [1]–[3], [5]. Comparisons based on global datasets are highly successful at elucidating large scale, biota-wide patterns; however, included data are subject to error due to stratigraphic biases [7], geographic incompleteness [8], and taxonomic inaccuracies [9] which limits the ability of these datasets to resolve detailed patterns. In particular these datasets do not include phylogenetic information and lack the detailed taxonomic, temporal, and evolutionary data required to investigate speciation-level processes, such as those potentially driving biodiversity loss during the Late Devonian Biodiversity Crisis. Analyzing species-level patterns within a well-constrained phylogenetic framework ameliorates some of these biases because the entire evolutionary history of an individual clade is examined. This is particularly true for clades in which a high percentage of their diversity is represented in the phylogenetic analysis [10]–[11]. Further, species-level analyses allow speciation mode to be assessed and thereby provide insight into macroevolutionary dynamics not possible with analyses based on higher taxa.

Speciation, the formation of new species from ancestral populations, occurs by two primary allopatric mechanisms: vicariance ( = allopatry model I of [12]), where the ancestral population becomes passively divided into two large subpopulations which subsequently diverge to form two daughter species, and dispersal ( = allopatry model II of [12], in which a small subset of the ancestral population actively migrates and diverges to form a new species [12]–[13]. Speciation mode at cladogenetic events can be discerned by examining phylogenetic topology within a biogeographic context [14]: episodes of vicariance are indicated by daughter species occupying only a subset of the ancestral range, whereas episodes of dispersal are indicated by daughter species occupying geographic areas different from or additional to the ancestral range.

Another notable feature of Middle to Late Devonian transition is a reduction in endemism, which has been related to the increased frequency of interbasinal species invasions at this time [15]–[18]. The increased amount of interbasinal species invasions potentially impacted opportunities for speciation. In analyses of speciation mode in modern organisms, speciation by vicariance occurs in much higher frequencies than speciation by dispersal [12], [19]–[21], a pattern which is repeated in fossil marine invertebrates [22]–[26]. In this analysis, the hypothesis that speciation rate declined during the Late Devonian biodiversity crisis due to a reduction in speciation by vicariance is tested.

Herein rates of speciation, extinction, and biodiversity turnover are calculated for three mainly North American clades: one bivalve subgenus, *Leptodesma (Leiopteria)*, and two brachiopod clades, *Floweria* and *Schizophoria (Schizophoria)* based on species-level phylogenic hypotheses previously published in [27]–[28]. In addition, speciation mode (vicariance vs. dispersal) at individual cladogenetic events is assessed by combining biogeographic distributions with phylogenetic hypotheses for these three clades and the subclass Archaeostraca, a clade of predatory crustaceans [29]. These clades were selected in order compare speciation patterns in the numerically dominant taxa from nearshore to offshore marine environments including both the dominant invertebrate predators and benthos. Because these taxa were common members of Middle through Late Devonian biota, this cross phyla analysis is likely representative of Devonian shallow marine invertebrate dynamics in general. Analyses indicate a fundamental shift in both speciation rate and mode during the crisis interval.

## Results

### Biodiversity rates

**Figure 1:**
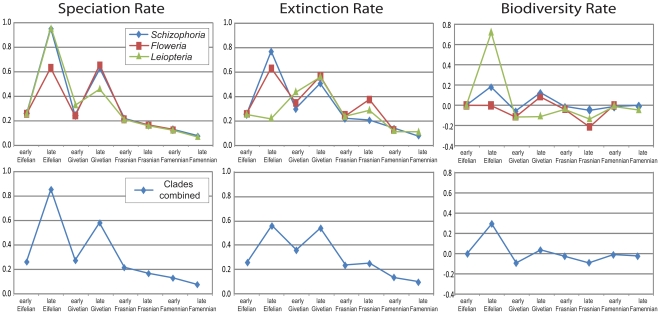
Instantaneous per-capita rates for speciation, extinction, and biodiversity change calculated using phylogenetically constrained species ranges (see Methods). The upper series displays rates calculated for each clade individually, and the lower series illustrates rates calculated with species of all clades combined. Biodiversity increases in the late Eifelian and late Givetian are driven by high speciation rates which overcome the effect of the high extinction rates. Extinction rates during the Frasnian biodiversity crisis interval are lower than during the Middle Devonian; however, biodiversity declines due to the severe reduction in speciation rates.

Biodiversity rates were calculated in three ways: per-capita rates based on observed species ranges, phylogenetically constrained per-capita rates, and phylogenetically constrained deterministic rates (see Methods). Results from the three sets of analyses produced congruent patterns (Table S1, Figs. 1, Figure S2). For clarity, the discussion below focuses on the phylogenetically constrained per-capita rates unless otherwise noted.

Figure 1 illustrates the phylogenetically constrained instantaneous per-capita rates of speciation, extinction, and biodiversity change calculated for each temporal bin spanning the interval prior to, including, and following the late Frasnian crisis interval. Although each clade exhibits some individuality of pattern, a congruent general pattern emerges. Biodiversity increased in the Middle Devonian, specifically during the late Eifelian and late Givetian intervals, followed by a continuous decline during the Frasnian with maximum biodiversity loss during the late Frasnian coinciding with the Late Devonian Biodiversity Crisis. Examining the two components of diversity change separately indicates that elevated speciation rates drove the late Givetian diversity increase, whereas elevated extinction rates contributed to the early Givetian and early Frasnian declines. Although extinction rates were elevated during the late Frasnian relative to the early Frasnian and Famennian intervals, late Frasnian extinction rates do not exceed that observed during Middle Devonian intervals. Notably, speciation rates declined to near zero in all clades during the Frasnian. Because extinction rates during the Frasnian crisis interval do not exceed background rates, the dramatic biodiversity loss is attributable to anomalously low speciation rates not high extinction rates.

A potential concern with phylogenetically constrained speciation rates is that artificially high rates of speciation can be calculated during the early history of a clade due to reconstruction of temporally equivalent splits of sister taxa from cladogenetic nodes and associated backward extension of species ranges (10–11). This potential bias does not appear to impact the results obtained here because the highest rates of speciation occur in the same temporal bins (late Eifelian and late Givetian) regardless of whether observed species ranges or phylogenetically constrained data are used (Table S1, Figs. 1, Figure S2). The observed temporal trend in speciation rate, therefore, is a primary pattern of the data rather than an artifact of the methodology.

To further test whether biodiversity decline was due to reduced speciation rate rather than elevated extinction rate, average Middle Devonian and average Late Devonian rates were statistically compared. Mann-Whitney U tests confirmed that Late Devonian speciation rates were significantly lower than the Middle Devonian speciation rates in all clades as well as the combined data set (α = 0.05 in all comparisons). Late Devonian extinction rates were also significantly lower than extinction rates during the Middle Devonian at the α = 0.05 level for *Schizophoria, Floweria*, and the combined dataset, although this comparison is marginally insignificant for *Leiopteria* (α = 0.10).

**Figure 2:**
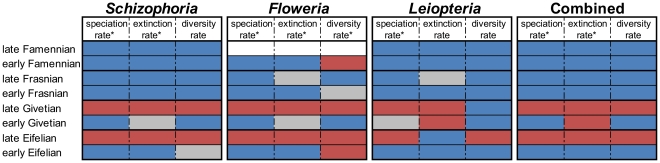
Comparison of instantaneous per-capita rates per interval versus the average rate across all intervals. Shading indicates directionality of rate comparison; blue indicates temporal bin rate lower than the average; red indicates temporal bin rate higher than the average; gray indicates approximately average rate. * indicates that rates during the Late Devonian intervals are significantly lower than rates during the Middle Devonian intervals based on the Mann-Whitney U-test. During Late Devonian, speciation rate is significantly lower than the average rate for all clades and extinction rate is either significantly lower or not significantly different than average rate. Biodiversity loss during the Frasnian crisis interval, therefore, is due to reduced speciation rates not elevated extinction rates.

The same pattern emerges if instantaneous rates for each temporal interval are compared to long-term rates for each clade. Specifically, instantaneous speciation and extinction rates were compared with the speciation/extinction rates characteristic of each clade throughout its duration as well as the average speciation/extinction rates for each clade for the entire study interval (Figs. 2, S3). Frasnian speciation rates were statistically lower than expected from the clade rate at the α = 0.05 level for all taxa. Extinction rates, although statistically elevated during the late Eifelian (α = 0.05), were not statistically higher than the clade rates during the late Frasnian, with the exception of *Floweria* in which rates are marginally higher (α = 0.01). These comparisons further support the argument that the primary driver of biodiversity loss during the Late Devonian biodiversity crisis was a dramatic decline in speciation rate.

### Speciation mode

The distribution of speciation events attributable to allopatric speciation by vicariance or dispersal for *Leptodesma (Leiopteria), Schizophoria (Schizophoria), Floweria*, and the crustacean suborder Archaeostraca are summarized in Table 1. Speciation events in *Schizophoria, Leptodesma*, and the archaeostracan crustaceans are overwhelmingly attributable to speciation by dispersal. Within these clades, speciation by dispersal is dominant, comprising 72% of events, whereas vicariance is only implicated in only 28% of speciation events (Table 1). Even in *Floweria*, where dispersal events were less common, the percentage of speciation events consistent with vicariance is lower than expected in comparison with modern and other fossil clades (Table 1).

**Table 1:** Comparison of allopatric speciation events by mode across Devonian, Early Paleozoic, and modern clades.

Clade	Number of vicariance events	Number of dispersal events	Percent speciation by vicariance	Percent speciation by dispersal
*Schizophoria (Schizophoria)*	2	17	11%	89%
*Floweria*	7	7	50%	50%
*Leptodesma (Leiopteria)*	2	6	25%	75%
Archaeostraca	6	13	32%	68%
**Devonian Combined**	17	43	**28% (sd 16%)**	**72% (sd 16%)**
**Cambrian and Ordovician Trilobites**			**54% (sd 16%)**	**46% (sd 16%)**
**Early to Middle Devonian Trilobites**			**54% (sd n/a)**	**46% (sd n/a)**
**Modern Fauna**			**74% (sd 35%)**	**26% (sd 34%)**

*Schizophoria (Schizophoria)* and *Floweria* are brachiopod clades, data from [28] *Leptodesma (Leiopteria)* is a bivalve clade, data from [27]. The subclass Archaeostraca is a phyllocarid crustacean clade, data from [29]. Early Paleozoic Trilobites comparison based on [23]–[26]; Middle Devonian trilobites based on [22] Modern comparison based on [20]. Devonian clades exhibit a dramatic reduction in speciation by vicariance when compared to the modern or other fossil taxa.

## Discussion

The combined results of the biodiversity rate analyses strongly indicate that the dramatic Frasnian speciation rate decline was the primary driver of biodiversity loss during the Late Devonian Biodiversity Crisis. This corroborates results of previous generic and family level analyses [3], [5], but temporal changes in rates alone cannot identify the biological reason for the decline in speciation rate. The analysis of speciation mode, however, does identify a mechanism for the observed decline in speciation rate: a dramatic lack of vicariant speciation during the study interval. In fact, all but two of the documented vicariance events precede the Frasnian Stage.

The observed level of Late Devonian vicariance is greatly reduced compared to that observed in modern and other fossil clades, in which the great majority of allopatric speciation occurs via vicariance (Table 1) [12], [19]–[21]. Most of the modern comparative data are based on analyses of continental taxa [12], [20], [30], which have been suggested to potentially exhibit higher levels of vicariance than marine taxa [31]. Recent speciation mode analyses of modern marine invertebrates, however, also document a 2∶1 ratio of speciation by vicariance versus dispersal [21], [32]–[33] indicating that vicariance is also the dominant speciation mode in modern marine ecosystems. Fewer studies have examined speciation mode in fossil marine invertebrates; however, several studies of Cambrian and Ordovician trilobites [23]–[26] also documented relative levels of vicariant speciation that greatly exceed those of the of the Late Devonian clades (Table 1). Significantly, the results of the present analysis indicate levels of Late Devonian vicariance that are substantially lower than that observed in Early to Middle Devonian trilobites [22] (Table 1). Devonian calmoniid trilobites of the cool-water Malvinokaffric realm also exhibited declining speciation rates associated with increased dispersal events as the Devonian progressed [34], which suggests the pattern reported herein is not restricted to Laurentia.

**Figure 3:**
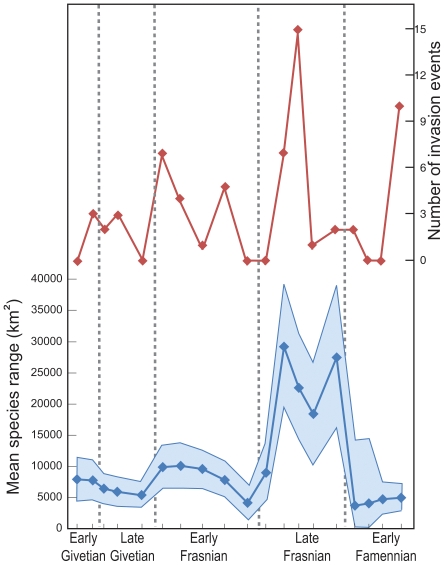
Middle to Late Devonian species geographic range sizes and invasion intensity. Both the mean geographic range size for brachiopod and bivalve species and interbasinal invasion intensity increase during Frasnian crisis interval compared with Givetian background levels. Modified from [18].

The Late Devonian reduction in vicariance coincided with increased intensity of interbasinal species invasions and expansion of species' geographic ranges [18] (Fig. 3). Interbasinal exchange was facilitated by multiple transgressive events that flooded previously emergent tectonic barriers within Laurentia [18], [35]. The establishment of broadly-adapted geographically widespread invasive species was the likely trigger for speciation depression. Allopatric speciation by vicariance requires passive isolation of previously adjacent populations [13]. The numerous range expansion events during this interval would have prohibited sustained geographic isolation, thereby cutting off the primary mechanism of vicariant speciation. Because vicariant speciation is the dominant speciation mode at other intervals of geologic time, halting vicariance would have resulted in a substantial lowering of speciation rate.

Speciation via dispersal, although more common than vicariance throughout the entire study interval, also stopped during the late Frasnian to Famennian interval. Analyses of the geographic ranges of species across the Late Devonian crisis interval have previously demonstrated preferential survival of species with large geographic ranges, an episode of interbasinal invasion in their history, and/or expanding geographic ranges during the late Frasnian [18], [36]. As species with small ranges were more likely to become extinct during the Late Devonian [18], the reduction in range size from an ancestral range to an isolated population required for speciation by dispersal would have been more likely to result in extinction than promote speciation during this interval.

By reducing opportunities for both vicariance and dispersal during the crisis interval, the effective speciation rate declined far beneath the marginally elevated extinction rate. This resulted in the tremendous loss of biodiversity and ecosystem overturn that characterizes the Late Devonian as no new species were forming to fill ecological voids left by the continuing extinctions. Combining these results with those of [18], [36], a more complete understanding of the faunal dynamics of the Late Devonian Biodiversity Crisis begins to emerge. Species that originated via dispersal events and participated in later episodes of interbasinal invasion were the species that successfully survived the biodiversity crisis interval. Conversely, ecologically specialized species with narrow geographic ranges became extinct without producing daughter species because of the general shutdown of vicariant speciation during this interval. This differential speciation and survival was manifested as a transition from the endemic faunas of the Middle Devonian to the cosmopolitan fauna of the Late Devonian and a shift from numerous more narrowly adapted species to fewer generalist species at the ecosystem level and higher.

Species invasions in the fossil record provide useful analogs for modern invasive species. These fossil invasions record the introduction of species into a tectonic basin outside of its native range and, therefore, into ecosystems in which they did not evolve [37]. This is identical in principle to modern human mediated introductions; therefore, modern invasive species should be expected to have similar impacts as ancient invaders. If vicariance is the more frequent mode of speciation, as indicated by studies of modern taxa [19]–[21], then the modern spread of invasive species may be expected to similarly result in speciation depression in modern ecosystems. Furthermore, if the Late Devonian is an accurate analog for modern ecosystems, modern human-mediated species invasions [38]–[40] should result in a similar long term diversity decline due to preferential survival of broadly adapted invasive species, extinction of geographically restricted ecological specialists, and suppression of vicariant speciation of new species.

## Materials and Methods

### Stratocladogram construction

In order to calculate speciation rates and assess speciation modes, a robust and detailed phylogenetic framework is required in order to determine evolutionary relationships and timing of cladogenetic events [41]–[42]. Without a phylogenetic framework, to ensure monophyly of species, paraphyletic or polyphyletic taxa may be included within rate calculations which could produce substantial errors in both absolute rates and the determination of temporal trends in relative rates [9], [20], [43].

Previously published species-level phylogenetic hypotheses for *Leptodesma (Leiopteria*) [27], *Floweria* [28], and *Schizophoria (Schizophoria)* [28] were converted to stratocladograms following the protocol of [41] (Fig. S1). Species temporal durations were reconstructed using known stratigraphic ranges, assuming sister taxa diverged simultaneously from cladogenetic nodes, and creating ghost ranges to connect theoretical speciation events with known range data. Species ranges span the late Emsian (Early Devonian) to Tournaisian (Early Mississippian) Stages. This interval was subdivided into ten temporal bins, one corresponding to the either early or late halves of each stage. The absolute duration of each stratigraphic bin was determined from the composite Devonian timescale of [44]. This timescale incorporates the greatest number of radiometric dates of any Devonian timescale and is interpolated against a biochronographic relative scale; consequently it represents the most robust hypothesis of absolute ages currently available for the Devonian Period.

### Calculation of biodiversity rates per temporal interval

Rates of instantaneous biodiversity change, speciation, and extinction were calculated from this data for each clade individually and for all three clades combined during each of ten temporal bins. Rates were calculated using three variants of the deterministic exponential model of taxon growth, which facilitates calculating instantaneous rates of biodiversity change within a phylogenetic framework [45]–[47].

Various speciation and extinction metrics have been proposed for rate calculation of modern and fossil taxa. Those most commonly applied to palaeontological data incorporate parameters related to standing diversity and absolute duration of temporal bins (see review in [48]); however, these typically utilize multi-taxon datasets derived from databases rather than single clades with known phylogenetic structure. Conversely, neontological methods of diversity rate analysis typically consider phylogenetic structure for clades, but produce a single value of speciation rate for the entire radiation of a clade rather than a rate per time interval [46]. Because this study combines both phylogenetic and temporal information, rates were calculated using both traditional paleontological rate estimation metrics designed to calculate instantaneous rates normalized by standing diversity (e.g., those of [48]), referred to herein as per-capita rates, and rate estimation methods designed to incorporate phylogenetic structure (e.g., those of [45], [47]), referred to herein as deterministic rates. Comparison of calculated rates provides a broader basis for interpreting the generality of the recovered biodiversity patterns. Each of metrics has a long history of use in phylogenetically-informed analyses of speciation and extinction rates [6], [34], [42], [49] or database-based estimates of biodiversity change [7], [9], [50]–[51].

Per-capita rates for speciation (qˆ), extinction (pˆ), and diversity change (d) were calculated following the equations in [48]. Rates were calculated for each clade individually and all clades combined for each temporal bin using counts from (1) raw species ranges and (2) phylogenetically corrected species ranges obtained from the stratocladograms in Figure S1 (Table S1). Values calculated from bins 1 and 10 were excluded from further analysis to remove edge effects.

Deterministic rates of biodiversity change (*R)*, speciation rate (*S*), and extinction rate (*E*) were calculated for each clade during each temporal bin based on phylogenetically corrected species range data in Figure S1 (Table S1). Rates are calculated as: *R* = (ln *N*_1_–ln *N*_0_)/Δ*t*; *S* = (ln (*N*_0_+*o*_0_)–ln *N*_0_)/Δ*t*; and *E* = (ln (*N*_0_+*o*_0_)–ln *N*_1_)/Δ*t*; where *N*_0_ is the initial number of species in a clade at time *t*_0_, *N*_1_ is the number of species present at time *t*_1_, Δ*t* is the duration of the interval *t*_1_–*t*_0_, and *o*_0_ is the number of speciation events during interval *t*_1_–*t*_0_ [48]–[49]. Following [52], the variance for each rate (*R*, *B*, or *D*) was calculated as: variance (*R*, *B*, or *D*) = [rate value (*R*, *B*, or *D*)]^2^/(*n*-2), and the standard deviation for each rate (*R*, *B*, or *D*) was calculated as: standard deviation (*R*, *B*, or *D*) = √[variance (*R*, *B*, or *D*)], where *n* = *N*_0_+*o*_0_, the number of species extant during the interval, [52]. Ninety-five percent confidence intervals were determined for calculated rate values based on the variance calculated using: 95% CI =  rate value (*R*, *B*, or *D*) ±2× standard deviation (*R*, *B*, or *D*). Values calculated from bins 1 and 10 were excluded from further analysis to remove edge effects.

### Calculations of general biodiversity rates per clade

Average per-capita speciation, extinction, and diversity change rates were calculated for each clade and for the combined dataset to provide a framework for comparing the instantaneous rates with rates throughout the Middle to Late Devonian study interval (Table S1). In addition, overall clade rates, rates exhibited by the clade during the entire duration of the study interval, were calculated using a pure-birth deterministic model based on species longevity following the principles outlined in [53]–[54] (Table S2).

To calculate clade rates, each species temporal range was interpreted to include the entire duration of all temporal bins in which that species occurs; range extensions of ghost ranges inferred from equal taxonomic split were not included. Species which range into the Mississippian were also excluded in order to calculate a rate characteristic of Devonian dynamics only. Because phylogenetic topology was not incorporated into longevity estimates, clade rates provide rate calculations independent from the phylogenetically corrected instantaneous biodiversity rates. The deterministic model assumes constant rates of speciation, extinction, and biodiversity change during the history of a clade that experiences exponential growth. Clade rates most accurately estimate biodiversity dynamics during the exponential growth or decay a clade from a starting clade size of one species [53], which is appropriate for the taxa examined herein.

The calculated of rate of biodiversity increase (R) follows the deterministic model outlined above assuming an initial standing diversity of one species as follows: R = (ln *n*)/Δ*t*, where *n* is the total number of species in the clade and Δ*t* is the entire temporal duration of the clade. Following [46], the standard deviation of R is calculated as: Standard deviation (R) = √(R^2^/(*n*-2)). Extinction rate (E) is calculated as the inverse of the longevity (temporal duration) of a species in the clade such that: E = 1/d, where d is the average species duration. The standard deviation of E is calculated as: Standard deviation (E) = √(1/var (d)). Following the same relationship between net biodiversity rate, speciation rate, and extinction rate described above, speciation rate (S) is calculated as: S = R+E. The standard deviation of S is calculated as: Standard deviation (S) = √((R^2^/(*n*-2)) +1/var (d)).

### Rate comparisons

Temporal variations in speciation, extinction, and diversity rates are presented graphically (Figs. 1, Figure S2). The rate analyses congruently indicate a reduction in both speciation and extinction rate during the Frasnian stage when compared to the Givetian stage. Several rate comparisons were conducted to determine whether the instantaneous speciation or extinction rates for individual temporal bins were, in fact, statistically different than expected under the null hypotheses that Late Devonian speciation and extinction rates were equivalent to or higher than Middle Devonian rates.

Per-capita rates for each interval were compared with (1) the average per-capita rate calculated across all temporal bins and (2) the clade rate. Instantaneous rates were coded as higher, lower, or equivalent to the average rates or the clade rate +/− 2 standard deviations (Figs. 2, S3). The statistical significance of the comparison between instantaneous per-capita rate and average per-capita rate was determined by comparing the four Middle Devonian intervals versus the four Late Devonian intervals using the non-parametric Mann-Whitney U test using the Bonferroni correction for multiple comparisons. Comparisons were conducted using both raw data and phylogenetically corrected data.

Instantaneous deterministic rates were compared with clade rates using 2-sample T-tests by incorporating calculated rate and standard deviations values for both values (Fig. S3).

### Speciation mode determination

To determine speciation mode at individual cladogenetic events, biogeographic areas were optimized onto internal nodes in the most parsimonious cladograms using the Fitch Parsimony algorithm, which provides a framework to interpret speciation mode at individual speciation events [14], [55]. Speciation mode was determined from taxon area cladograms for the three clades described above and the crustacean Subclass Archaeostraca [27]–[29]. Each node on the optimized taxon-area cladograms was examined for evidence of speciation by vicariance or dispersal following criteria of [12], [14]. Biogeographic shifts between ancestor and descendant taxa were identified as due to *vicariance* at nodes where descendant taxa occupy only a subset of the ancestral range or *dispersal* at nodes where descendant taxa occupy geographic regions additional to or different from the ancestral range.

## Supporting Information

Figure S1 **Stratocladograms.** Species-level phylogenetic hypotheses from [26]-[27] modified into stratocladograms. Solid lines indicate a species observed range, while dashed lines indicate ghost lineage range extensions. Absolute age dates and relative time scale modified from [44].
(TIF)

Figure S2 **Additional speciation, extinction, and biodiversity rates.** Upper two series illustrate instantaneous deterministic rates calculated using phylogenetically constrained species ranges by clade (top row) and for all three clades combined (second row). Vertical bars indicate 95% confidence intervals. Lower two series illustrate instantaneous per-capita rates for speciation, extinction, and biodiversity change calculated from raw species range data by clade (third row) and for all three clades combined (fourth row). Instantaneous per-capita rates could not be calculated for *Leiopteria* in any interval or *Schizophoria, Floweria*, or the combined data sets in select intervals due to values of 0 in the data distribution (Table S1). Regardless of rate calculation method, all analyses indicate a significant decline in speciation rate during the Frasnian coupled with extinction rates that are not elevated beyond those of the Middle Devonian during the crisis interval.
(TIF)

Figure S3 **Comparison of instantaneous rates versus clade or average rates.** Calculated rates for individual temporal bins are compared with the clade rate or average of the rate during the study interval. 95% confidence intervals were constructed for both clade rates and deterministic rates (see Methods) but not per-capita rates and average rate values. If calculated per-capita rates are above the 95% CI for the clade rate or above the average rate, the box is shaded red, rates that fall below were shaded blue, and those that are indistinguishable were coded gray. Shading of the deterministic rate versus clade rate indicates statistical significance of T-test comparison; blue indicates temporal bin rate significantly lower than the clade rate (p<0.05); red indicates temporal bin rate significantly higher than the clade rate (dark red: p<0.05, light red: p<0.01). Results of all five comparisons are highly congruent. Both speciation and extinction levels are low in the Late Devonian relative to Middle Devonian values.
(TIF)

Table S1 **Rate calculations.** Instantaneous per-capita and deterministic rates calculated as described in Methods. Raw observed turnover data includes only the observed stratigraphic ranges of species while phylogenetically constrained data incorporates ghost ranges as outlined in Methods.
Rates are calculated from species turnover data to the left of the rate columns. Δ*t* is the temporal duration of the interval determined from [44] (Fig. S1). N_bL_ indicates the number of species that cross the lower interval boundary but become extinct during the interval, N_Ft_ indicates the number of species that originate within the interval and cross over the upper interval boundary, N_bt_ indicates the number of species that cross both the upper and lower interval boundaries. *N_0_* indicates the number of species extant at the beginning of the interval *t_n_*, *N*_f_ indicates the number of species extant at the end of interval of time *t_n_,* # sp indicates number of speciation events that occur during that interval, # ext indicates the number of species that become extinct during the interval. Rate and standard deviation values derived from equations in Methods.
(XLS)

Table S2 **Clade rates.** Clade rates are calculated from the deterministic equation described in Methods for each clade during the Devonian and all clades combined during the study interval.
(XLS)
