# Supplementary figures and images for: Invasive Species and Biodiversity Crises: Testing the Link in the Late Devonian
Source: PLoS One. 2010 Dec 29;5(12):e15584. doi: 10.1371/journal.pone.0015584 (PMC3012059; doi:10.1371/journal.pone.0015584)

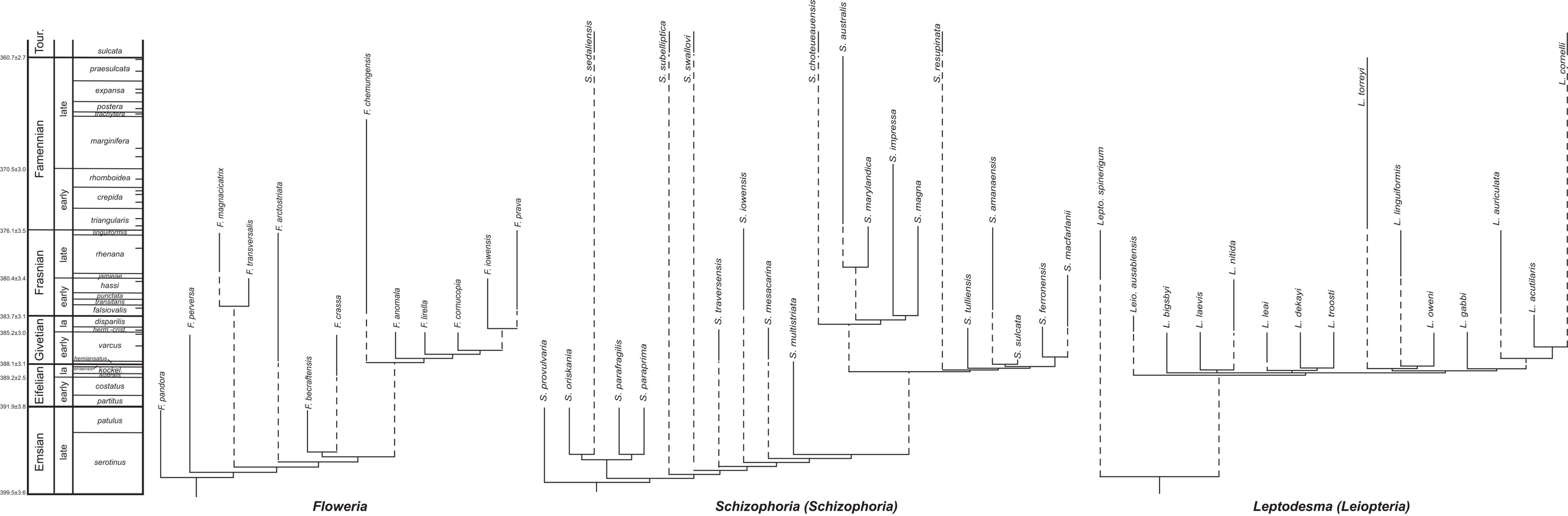

Supplement: Figure S1 — Stratocladograms. Species-level phylogenetic hypotheses from [26]-[27] modified into stratocladograms. Solid lines indicate a species observed range, while dashed lines indicate ghost lineage range extensions. Absolute age dates and relative time scale modified from [44]. (TIF) [file pone.0015584.s001.tif]

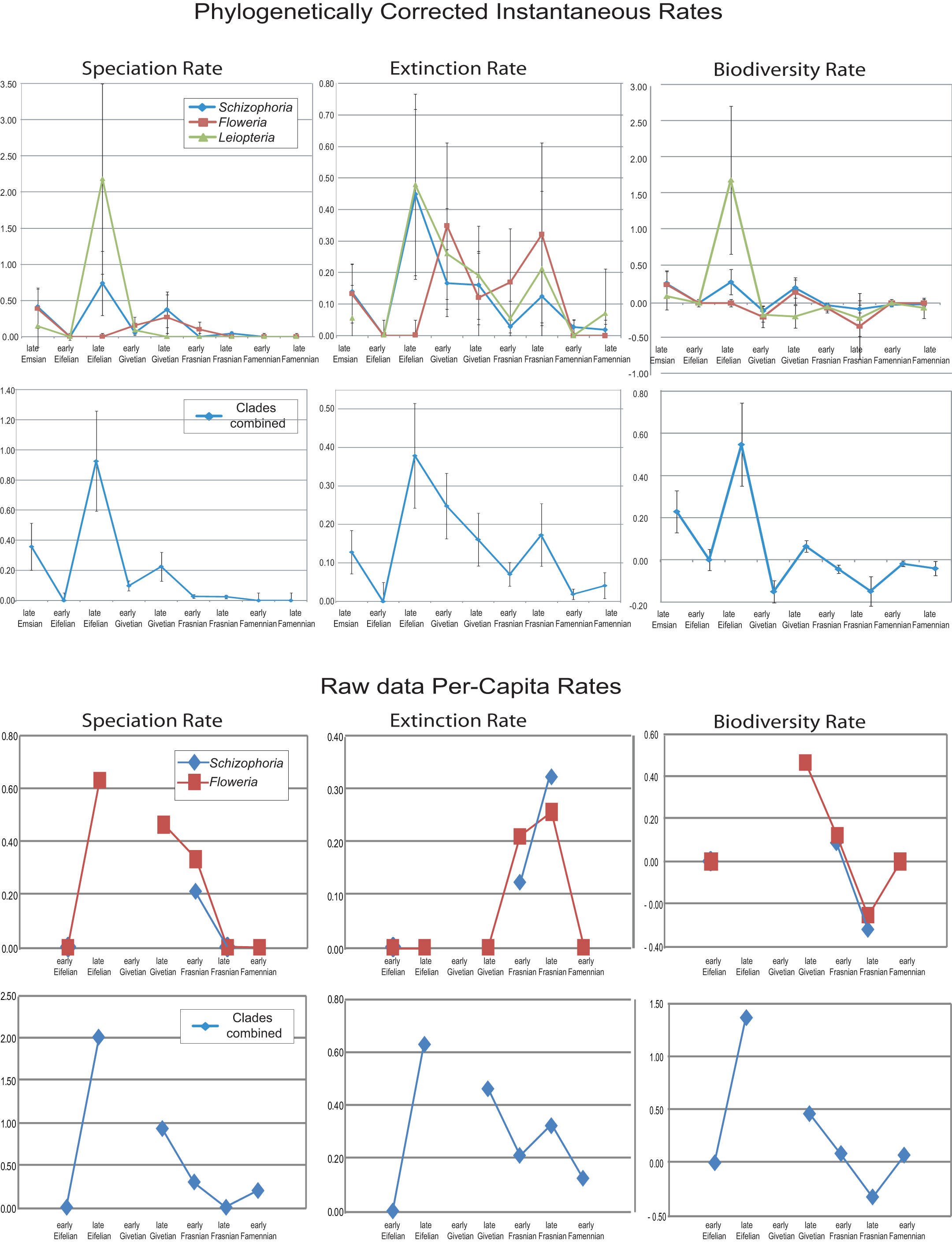

Supplement: Figure S2 — Additional speciation, extinction, and biodiversity rates. Upper two series illustrate instantaneous deterministic rates calculated using phylogenetically constrained species ranges by clade (top row) and for all three clades combined (second row). Vertical bars indicate 95% confidence intervals. Lower two series illustrate instantaneous per-capita rates for speciation, extinction, and biodiversity change calculated from raw species range data by clade (third row) and for all three clades combined (fourth row). Instantaneous per-capita rates could not be calculated for Leiopteria in any interval or Schizophoria, Floweria, or the combined data sets in select intervals due to values of 0 in the data distribution (Table S1). Regardless of rate calculation method, all analyses indicate a significant decline in speciation rate during the Frasnian coupled with extinction rates that are not elevated beyond those of the Middle Devonian during the crisis interval. (TIF) [file pone.0015584.s002.tif]

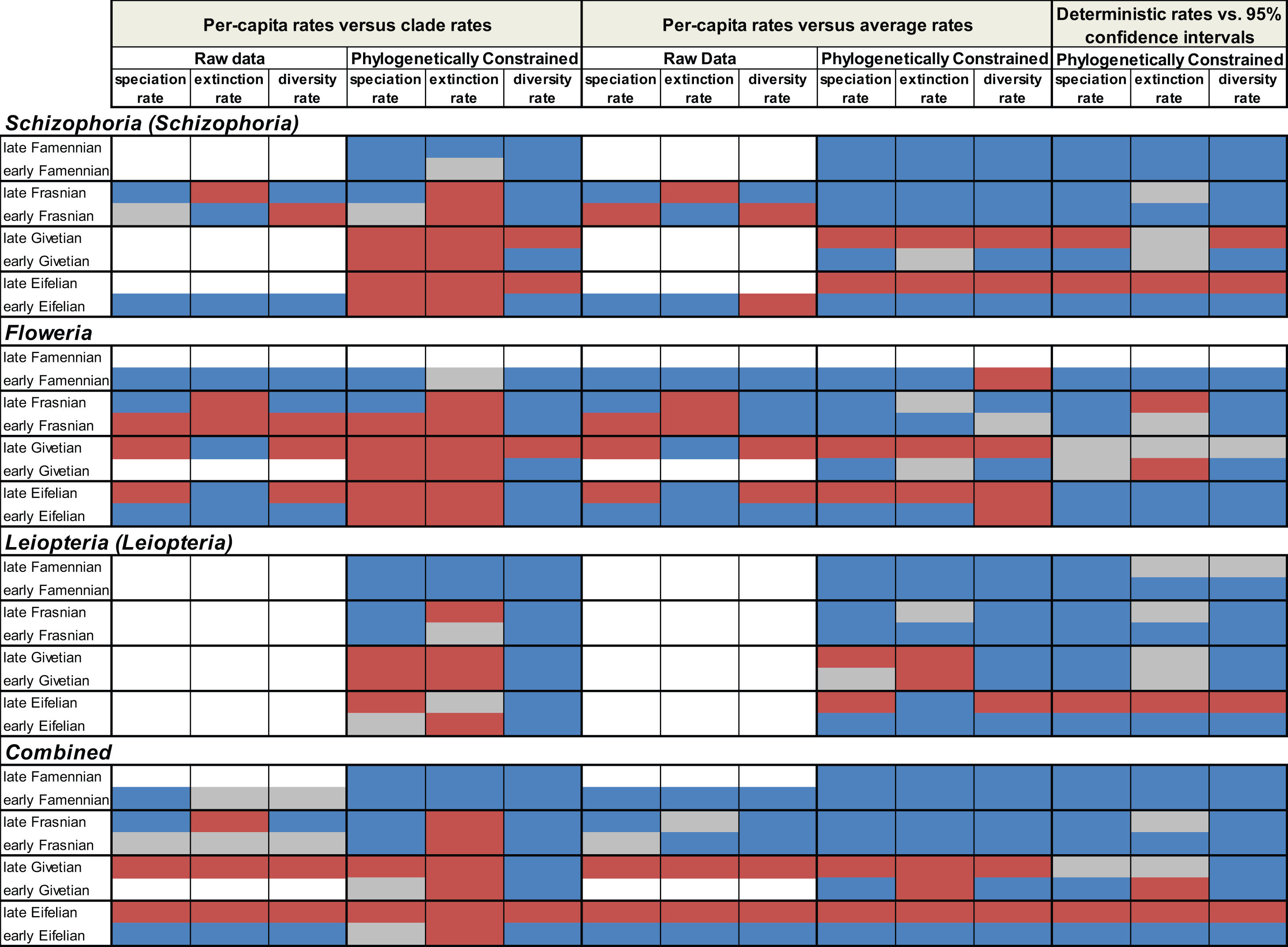

Supplement: Figure S3 — Comparison of instantaneous rates versus clade or average rates. Calculated rates for individual temporal bins are compared with the clade rate or average of the rate during the study interval. 95% confidence intervals were constructed for both clade rates and deterministic rates (see Methods) but not per-capita rates and average rate values. If calculated per-capita rates are above the 95% CI for the clade rate or above the average rate, the box is shaded red, rates that fall below were shaded blue, and those that are indistinguishable were coded gray. Shading of the deterministic rate versus clade rate indicates statistical significance of T-test comparison; blue indicates temporal bin rate significantly lower than the clade rate (p<0.05); red indicates temporal bin rate significantly higher than the clade rate (dark red: p<0.05, light red: p<0.01). Results of all five comparisons are highly congruent. Both speciation and extinction levels are low in the Late Devonian relative to Middle Devonian values. (TIF) [file pone.0015584.s003.tif]
